# Presence of Native Prey Does Not Divert Predation on Exotic Pests by *Harmonia axyridis* in Its Indigenous Range

**DOI:** 10.1371/journal.pone.0159048

**Published:** 2016-07-08

**Authors:** Gui Fen Zhang, Gábor L Lövei, Xia Wu, Fang Hao Wan

**Affiliations:** 1 State Key Laboratory for Biology of Plant Diseases and Insect Pests, Institute of Plant Protection, Chinese Academy of Agricultural Sciences, Beijing, China; 2 Department of Agroecology, Aarhus University, Flakkebjerg Research Centre, Slagelse, Denmark; 3 College of Agronomy and Plant Protection, Qingdao Agricultural University, Qingdao, Shandong, China; French National Institute for Agricultural Research (INRA), FRANCE

## Abstract

In China, two invasive pests, *Bemisia tabaci* MEAM1 (Gennadius) and *Frankliniella occidentalis* (Pergande), often co-occur with the native pest, *Aphis gossypii* (Glover), on plants of Malvaceae and Cucurbitaceae. All three are preyed on by the native ladybird, *Harmonia axyridis* (Pallas); however, the native predator might be expected to prefer native prey to the exotic ones due to a shared evolutionary past. In order to clarify whether the presence of native prey affected the consumption of these two invasive species by the native predator, field-cage experiments were conducted. A duplex qPCR was used to simultaneously detect both non-native pests within the gut of the predator. *H*. *axyridis* readily accepted both invasive prey species, but preferred *B*. *tabaci*. With all three prey species available, *H*. *axyridis* consumption of *B*. *tabaci* was 39.3±2.2% greater than consumption of *F*. *occidentalis*. The presence of *A*. *gossypii* reduced (by 59.9% on *B*. *tabaci*, and by 60.6% on *F*. *occidentalis*), but did not stop predation on the two exotic prey when all three were present. The consumption of *B*. *tabaci* was similar whether it was alone or together with *A*. *gossypii*. However, the presence of aphids reduced predation on the invasive thrips. Thus, some invasive prey may be incorporated into the prey range of a native generalist predator even in the presence of preferred native prey.

## Introduction

Problems associated with biological invasions, both environmental and economic, have grown exponentially over the past decades [1]. Invasive species threaten natural habitats and can affect native organisms in various ways [2] and at different levels of biological organization [3,4] typically because predation pressure on the invader from natural enemies is relaxed [5], as well as due to the potential disruption of trophic interactions between local prey and local enemies [6]. Native natural enemies are one factor in the 'biotic resistance' concept of invasion biology [7], but it is unknown how many invasion episodes fail due to such activity. The activity of native natural enemies, in the form of biological control, is usually considered a powerful component of invasive species management, and 'classical' biological control, the use of exotic, specialist natural enemies to control exotic pests, has a long history [3]. Biological control using exotic generalist natural enemies, however, is generally not advisable because they may pose a threat to non-target native species [3]. However, native generalist predators can play an important role in the control of new invaders [3,8–14].

With China's increasing involvement in world trade since the 1970's, the number of invasive species originating from China, as well as the number arriving, have increased substantially [15]. The estimated rate of new arthropod pests establishing in China is 14 species per decade [15]. Two groups among them, whiteflies (Aleyrodidae) and thrips (Thysanoptera: Thripidae) have had large impacts on Chinese agriculture. Currently, the most important non-native whitefly is *Bemisia tabaci* (Gennadius) MEAM1 (Hemiptera: Aleyrodidae), a member of the silverleaf whitefly species group [16]. This species originates in the Middle East and Asia Minor [17,18] and invaded China in the late 1990’s [19]. Another important invader is the western flower thrips, *Frankliniella occidentalis* (Pergande), a species native to western North America [20]; it was first recorded in China in 2003 [21]. Both species are important pests of agricultural and horticultural crops in many of the regions they have invaded [22–24]. The cotton aphid (also called melon aphid), *Aphis gossypii* (Glover) (Hemiptera: Aphididae), is also an important pest of numerous crops [25] and is considered a native species in China, although it has a worldwide distribution.

Plant protection problems are often exacerbated because these pests have all evolved high levels of insecticide resistance (for *B*. *tabaci* MEAM1, see [26,27]; for *F*. *occidentalis*, see [28]; and for *A*. *gossypii*, see [29,30]). Natural enemies play an important role in regulating pest populations [31], including generalist predators [32,33], and many of them are important in biological control. The multicolored Asian lady beetle, *Harmonia axyridis* (Pallas) (Coleoptera: Coccinellidae), is native to central and eastern Asia [34] and has been used as a biological control agent against aphids worldwide [35]. This ladybird beetle can also feed on scale insects, thrips, whiteflies and other soft-bodied arthropods [36,37].

Of the potential prey of *H*. *axyridis*, *B*. *tabaci* MEAM1 and *F*. *occidentalis* co-occur in greenhouses and open fields in northern and eastern China, sometimes with a simultaneous presence of *A*. *gossypii* on several plant species in the Malvaceae, Cucurbitaceae and Rosaceae families [38]. They are all preyed on by *H*. *axyridis* larvae and adults (GF Zhang personal observation), but the efficacy of this species against the invasive *B*. *tabaci* MEAM1 and *F*. *occidentalis* remains unknown, especially in the presence of the native *A*. *gossypii*. Part of the problem is to detect if a single predator has fed on multiple prey species and how many prey individuals within a single predator [39]. DNA-based gut-content analysis is one method that has been applied in field studies to map trophic relationships (*e*.*g*. [8,9,40–42]) but usually with a focus on single prey species. Multiplex PCR systems, which allow simultaneous amplification of several DNA fragments within one reaction, are a more recent and promising method for addressing complex trophic interactions [41,43–46].

In this study, we examined multiple trophic linkages in *H*. *axyridis*, focusing on its consumption of two exotic pest species in the presence vs. absence of a native pest, the aphid *A*. *gossypii*. Due to a pre-existing evolutionary connection via a predatory-prey relationship, we hypothesised that the presence of sufficient quantities of this native prey would reduce, and possibly stop the consumption by *H*. *axyridis* of these exotic prey species. On the other hand, *H*. *axyridis* is a generalist predator, so may be pre-adapted to a wide range of prey, and it is also plausible to assume that predation on exotic prey species would not be totally disrupted by the presence of native prey.

## Materials and Methods

### Biological materials

Insect colonies of pest species were reared at the Department of Biological Invasions, Institute of Plant Protection, Chinese Academy of Agricultural Sciences (CAAS). A colony of *Bemisia tabaci* MEAM1 (henceforth Bt) was started from individuals collected on field-grown cabbage in the outdoor plots of the institute 5 years ago, and maintained on tomato plants (*Lycopersicon esculentum* L., cv. Zhongshu 5), under greenhouse conditions [47]. A colony of *F*. *occidentalis* (henceforth Fo) started with individuals collected on sweet pepper grown at the same location 4 years ago, and maintained on fresh kidney bean (*Phaseolus vulgaris* L., cv. Gongjizhe) pods in an insect rearing room [48]. A colony of *A*. *gossypii* (henceforth Ag) was started from individuals collected from outdoor cotton plants during the 2009 field season, and maintained on cucumber plants (*Cucumis sativus* L., cv. Zhongnong 18), under greenhouse conditions at 20–26°C and 60±10% RH with a 16 h:8 h light:dark photoperiod. Cucumber plants were cultivated in a mixed potting medium (soil, vermiculite and plant ash, 1:1:1) in plastic pots (130×175 mm in diameter), and were kept in a clean seedling greenhouse. The plants were checked daily and watered as needed. When the cucumber plants had 4–5 fully extended true leaves, they were put in whitefly- and thrips-proof screen cages (60×60×60 cm high, tent-like) (NingBoSafe Experimental Instrument Co., Ltd., Ningbo, China) for rearing the cotton aphid. Each cage contained eight plants. Every week, four plants were replaced with new ones.

Individuals of the predatory coccinellid *H*. *axyridis* were collected as newly formed pupae from maize fields near Beijing, immediately before the experiments, and kept under controlled conditions for about one week, under 25±2°C and 70±10% RH with a 8 h:16 h light:dark photoperiod. Once emerged, the adults had access to only 10% sucrose solution for 24–48 h before being used in the experiments.

### Field cage trials

In order to test if there is a difference in prey consumption depending on the nature and diversity of prey present, cages with one (Bt or Fo alone), two (Bt and Ag, Fo and Ag, Bt and Fo, respectively), three (Bt, Fo and Ag) prey species were used in field cage trial. Since our method (*i*.*e*. duplex TaqMan quantitative PCR [qPCR]) did not allow us to quantify consumption of *A*. *gossypii*, there was no cage with only Ag. The field cage trials were conducted at the Experimental Station of CAAS, Langfang, Hebei, China (39°30’36.96”N, 116°36’31.87”E) during the 2010 growing season. Cucumber plants were grown from seeds in plots protected from insect infestation by screen cages (2×2×2 m) and without application of pesticides. Each cage contained 20 cucumber seedlings, planted at 39–41 cm × 34–36 cm plant distances in 4 rows by 5 columns, facing the south. Distances between seedlings and cage edges were ~40 cm. Plants were checked daily and watered as needed. When the cucumber plants had 4–5 fully expanded true leaves, we established the following combinations, each in triplicate. Moreover, because of possible mortality after transfer, the densities of the exotic and native prey species had to be adjusted one week later. Since about 10–20% Fo adults rest in crevices, count of about 80–90% adults indicated that all adults were present (GF Zhang personal experience).

#### Bt alone

About 100 Bt adults / plant (sex ratio = 1:1; 3–5 d old; sexed under a stereomicroscope, 180× magnification) were released in each cage. Seven days later, the density of Bt was adjusted to 100 adults / plant by adding or removing whiteflies as needed. There were 50–70 eggs / plant at this time.

#### Fo alone

About 100 Fo adults (3–5 d old) / plant were released in each cage. The sex ratio was adjusted to 2:1 (female:male) as in nature; individuals were sexed under a stereomicroscope (180× magnification). Seven days later, the density of Fo was adjusted to 90–100 adults / plant by adding or removing adults as needed. At this stage, there were also about 30 eggs in tender tissues and about 30 1st-2nd nymphs / plant.

#### Bt and Ag

About 50 Bt adults / plant (sex ratio = 1:1) and 30 Ag adults / plant were released in each cage. Seven days later, the densities were checked and adjusted. Bt density was adjusted to 50 adults / plant by adding or removing whitefly adults, plus 20–30 eggs / plant. The density of Ag was adjusted to 50–60 adults / plant.

#### Fo and Ag

About 50 Fo adults / plant (sex ratio = 2:1) and 30 Ag adults / plant were released in each cage, and allowed to reproduce for 7 days, whereupon Fo density was adjusted to 40–50 adults / plant. In addition, there were 10–20 eggs in tender tissues and 10–20 nymphs (1st-2nd instar) per plant. The density of Ag was adjusted to 50–60 adults / plant.

#### Bt and Fo

About 50 adults / plant each of Bt (sex ratio = 1:1) and Fo (sex ratio = 2:1) were released in each cage. Seven days later, the density of Bt was adjusted to 50 adults / plant plus 20–30 eggs / plant and that of Fo to 40–50 adults / plant plus 10–20 eggs in tender tissues and 10–20 1st-2nd nymphs.

#### Bt and Fo and Ag

*B*. *tabaci*, *F*. *occidentalis* and *A*. *gossypii* (Bt—Fo—Ag): about 50 Bt adults / plant (sex ratio = 1:1), 50 Fo adults / plant (sex ratio = 2:1) and 30 Ag adults / plant were released in each cage. Seven days later, we adjusted prey densities to 50 Bt adults / plant plus 20–30 eggs, 40–50 Fo adults / plant plus 10–20 eggs in tender tissues and 10–20 1st-2nd nymphs, and 50–60 Ag adults / plant.

Eighty newly emerged *H*. *axyridis* adults were released into each cage. All the treatments and replications were conducted simultaneously. At given times (1h, 2 h, 4 h, 8 h, 12 h, 24 h and 36 h after predator release), 10 *H*. *axyridis* adults were collected using a mouth aspirator, individually put into single 1.5 mL microcentrifuge tubes filled with 95% ethanol, and kept at -40°C until analysis. We were concentrating on prey consumption and not on biological control effect, so the exact numbers of each prey species were not checked, only that there were still prey available at the end of the experiment. There were about 20 prey individuals left on each plant when the last sample was taken (36 h after predator release), so we consider that prey availability was *ad libitum*. Five randomly selected beetles were individually homogenized and tested for the presence of Bt and/or Fo in their guts for each time period from each field cage. The DNA of prey species in the predator gut was detected and quantified by using qPCR and external calibration (standard curve).

### DNA extraction

Bt or Fo adults were individually homogenized in 1.5 mL mortar-and-pestle microcentrifuge tubes (Haimen Sanhe Huasheng Glasses Materials Ltd., Jiangsu, China) in 200 μL of DNA extraction buffer supplemented with SDS to 1% and proteinase K to 20 mg mL^-1^ [46]. The homogenate was vortexed briefly and incubated in a water bath at 60°C for 1 h, with a re-mixing after 30 min. After incubation, the tubes were boiled for 5 min to inactivate the proteinase K, after which the lysate was extracted with 200 μL of chloroform-isoamyl alcohol mix (24:1, v/v), kept on ice for 30 min and centrifuged at 12 000 *g* for 20 min. This process was repeated twice, and the aqueous phase was mixed with 400 μL of ice-cold 100% ethanol. DNA was pelleted by centrifugation, dried and re-suspended in 20 μL ultra-pure water and stored at -40°C. The same DNA extraction procedure was used for non-target whitefly and thrips species (see section Specificity of the detection system), as well as for the predators collected from the field cage treatments. Before DNA extraction, individual predators were washed twice with ice-cold, pure water and blotted thoroughly with a clean filter paper, and the wings and legs were cut off.

### Primers and probes used for duplex TaqMan qPCR

Based on an established single tube, one-step duplex PCR [46], primers and probes for the Bt specific (Bt1 isolate) [9] and Fo specific (Fo1 isolate) (present study) DNA quantification were designed by using the PRIMER EXPRESS software (Applied Biosystems). The primers and probes (Table 1) were synthesized by Shanghai GeneCore BioTechnologies.

**Table 1:** Details of primer and probe sequences (5’-3’), fluorescent labels and expected product size for primers and probes used in duplex qPCR analyses.

Species	Name	Primer sequence 5’-3’	Size (bp)	Reference
Bt	FP-Bt1	TGTACTCGAAACCATTGATAGCTCA	93	[9]
	RP-Bt1	TTACTCGAGGTTGGCCGAAA		
	TaqMan-Probe 18953	VIC-CGCCCTGTCCTCCTA-MGB		
Fo	QZWF-Fo1	GGGAAGAAGAAGACTGCCACTATG	138	Present study
	QZWR-Fo1	GGGTCAGCAGGTGGAGTTTACT		
	TaqMan-Probe (FoQZWP)	FAM-ACCCACTTCATCTTGCT-MGB		

Two gene sequences from genomic DNA were simultaneously targeted in this duplex qPCR procedure. The first targeted sequence was a 93 bp long segment present only in Bt [9], and the second was a 138 bp segment specific to Fo (present study). The system and conditions used for the duplex TaqMan qPCR were optimized and performed with ABI PRISM 7500 Fast Real-Time PCR System (Applied Biosystems). Reactions were performed in a final total volume of 25 μL. Each tube contained 1.25 μL of each forward primer (20 pM μL^-1^), 1.25 μL of each reverse primer (20 pM μL^-1^), 0.625 μL of each fluorogenic probe (10 pM μL^-1^), 2.0 μL of each DNA template, 12.5 μL TaqMan Universal PCR Master Mix, 0.5 μL ROX Dye^TM^ and 1.75 μL ultra-pure water. Amplification conditions were: denaturation for 3 min at 95°C, 40 cycles of amplification (95°C for 15 s, 59°C for 1 min).

### Specificity of the detection system

To confirm the efficacy and specificity of the duplex qPCR, we used the following prey species, these are the most likely other species possibly present in the field especially those closely related which are more likely to present false positive for the specific primers, as negative controls [46]:

- two non-target, cryptic species of *B*. *tabaci* (Asia II 3 and Asia II 1),
- four non-target whitefly species (*Trialeurodes vaporariorum* [Westwood], *Dialeurodes citri* [Ashmead], *Aleurocanthus spiniferus* Quaintance [collected from orange trees in Yidu, Hubei, China in 2011] and *Aleurodicus dispersus* Russell),
- nine other thrips species, *Frankliniella intonsa* (Trybom), *Frankliniella tenuicornis* (Uzel), *Thrips flavidulus* (Bagnall), *Thrips tabaci* Lindeman, *Thrips hawaiiensis* (Morgan), *Thrips flevas* (Schrank), *Anaphothrips sudanensis* (Trybom), *Microcephalothrips abdominalis* (Crawford) (all Thysanoptera: Thripidae), and *Haplothrips aculeatus* (Fabricius) (Thysanoptera: Phlaeothripidae),
- the aphid *A*. *gossypii*, and
- starved *H*. *axyridis* adults.

In all instances, primers and probes against Bt and Fo showed positive fluorescent signals; but we obtained no positive fluorescent signal from any of the above species, indicating target specificity.

### Quantification of prey number consumed by *H*. *axyridis*

The duplex qPCR standard curves for Bt and Fo were generated using DNA from the purified plasmid Bt1 and Fo1 isolates originated from our laboratory. The DNA concentrations were determined by a NanoPhotometer P-Class (Implen, Munich, Germany) and the numbers of target DNA sequence copies were calculated using the expression:

$$\mathrm{number of target DNA copies}=\left(\mathrm{DNA mass}\;\left[\mu g\right]/\mathrm{DNA molar mass}\right)\times 6.023\times 10^{23}.$$

A volume of 2 μL was used as template in each duplex TaqMan qPCR assay. The qPCR standard curves were established using 10-fold serial dilutions of the number of target DNA copies of Bt (Bt1 isolate) and Fo (Fo1 isolate) from 9.958×10^6^ to 9.958×10^2^ and 1.217×10^7^ to 1.217×10^3^, respectively. Three repetitions of the assay were undertaken.

Data acquisition and analysis were performed with the ABI PRISM 7500 software. The values of threshold cycle (*C*_t_) correspondence to the five DNA copy numbers were 19.20±0.09, 22.47±0.05, 25.82±0.03, 28.95±0.06, 31.73±0.14 for Bt and 19.12±0.01, 22.55±0.13, 25.47±0.10, 28.57±0.10, 31.78±0.09 for Fo, respectively. When plotting respective *C*_t_ values against log_10_ of the numbers of Bt1 or Fo1 isolate copies per reaction, strong linear relationships were obtained for both Bt (*y* = - 3.1340*x* + 41.333; *r*^2^ = 0.9995) and Fo (*y* = - 3.1520*x* + 41.384; *r*^2^ = 0.9988), making the *C*_t_ value a reliable way to quantify DNA and estimate Bt1 and Fo1 isolate numbers. Numbers of Bt- and Fo- specific DNA copies from field-cage collected samples were estimated by extrapolation from the DNA standard curves amplified in the same PCR run. All samples generating fluorescence signals that exceeded a defined background threshold (*i*.*e*. *C*_t_ ≤ 35 in this study) were classified as positive [49]. The numbers of Bt and Fo adults consumed by each predator individual were calculated from the respective DNA copy numbers of individual adult of Bt (25 583.5±485.5) [9] and Fo (female, 36 692.8±4 068.4; Zhang *et al*. unpublished data) by using the above linear relationships and are reported as means and standard error (SEM).

### Data analysis

Percentage data, *i*.*e*. percentage of predator individuals that consumed Bt or Fo as detected by the molecular method, were arcsine transformed and the numbers of Bt and Fo adults were log-transformed before analysis of variance (ANOVA). The field-cage experiment and duplex qPCR analysis produced mean numbers of Bt and Fo adults and data on the percentage of predation by *H*. *axyridis* on Bt or Fo. Such data were subjected to two-way (treatment and time after predator release) and one-way (same time after predator release among different treatments, or same treatment among different time after predator release) ANOVA, and means were tested using Fishers LSD test [50]. The differences in same focal prey species among different treatments were compared by paired samples *t*-test. The numbers of Bt and Fo adults and percentage of predation by *H*. *axyridis* on Bt or Fo were reported as means and standard error (SEM). Since there were no significant differences in numbers of Bt and Fo adults consumed by *H*. *axyridis* among cages, the data of the three field cages were pooled and analyzed together.

## Results

### Predation on single prey species

**Fig 1:**
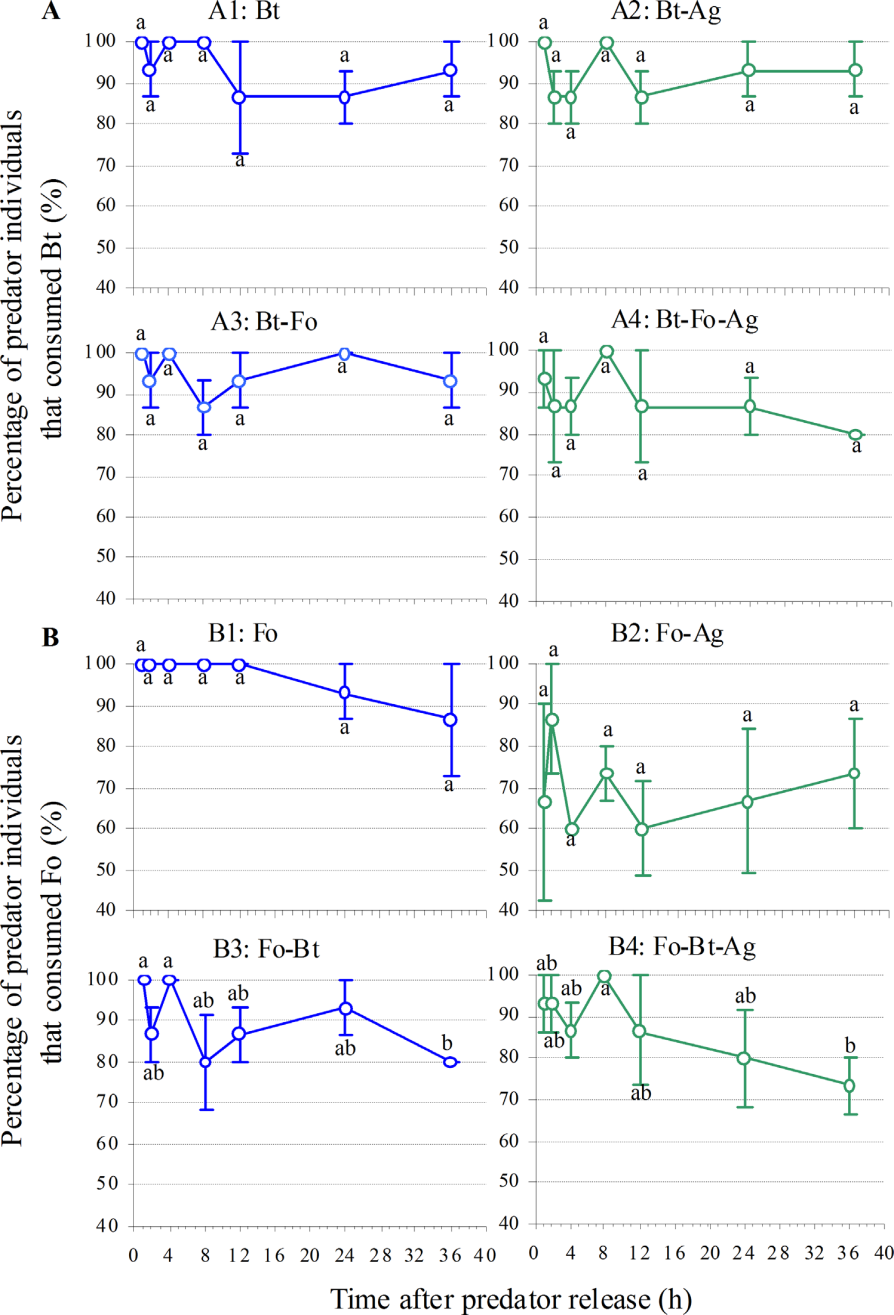
**Mean percentage of predator individuals (n = 3 cages; ±1 SE) that consumed (A) *Bemisia tabaci* MEAM1 (Bt) or (B) *Frankliniella occidentalis* (Fo) in various prey species combinations**. Means within a treatment followed by different lower case letters differ at *P* < 0.05 (one-way ANOVA, LSD test). Abbreviation: Ag—*A*. *gossypii*.

**Fig 2:**
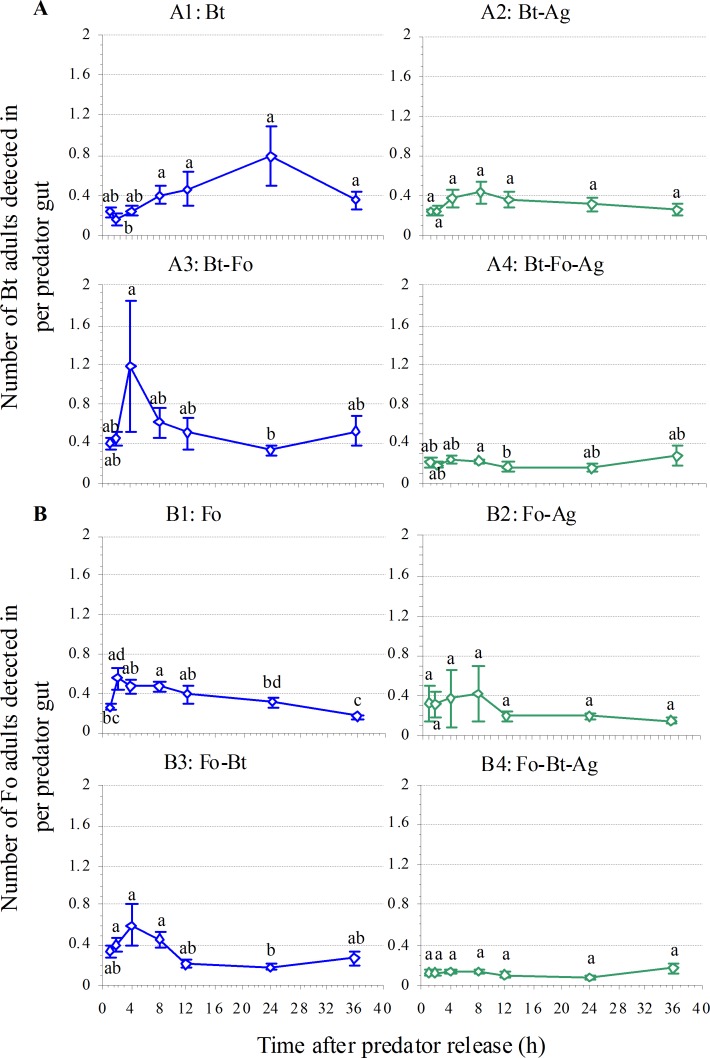
**Mean number of (A) *Bemisia tabaci* MEAM1 (Bt) or (B) *Frankliniella occidentalis* (Fo) adults detected in *Harmonia axyridis* gut (n = 15 ladybird beetles; ±1 SE) in various prey species combinations**. Means within a treatment followed by different lower case letters differ at *P* < 0.05 (one-way ANOVA, LSD test). Abbreviation: Ag—*A*. *gossypii*.

Adult ladybirds readily attacked both Bt and Fo in a no-choice situation. From 1 to 8 h after release, 90% of predators contained prey DNA; between 12 h and 36 h, a non-significant decrease to >80% was registered (Bt: *P* = 0.531, Fo: *P* = 0.557; Fig 1A_1_ and 1B_1_). The maximum of Bt prey was about 0.78 adults 24 h after predator release, while for Fo, a peak of 0.55 adults / predator was reached 2 h after predator release before a later decrease. Time after predator release (Bt: *P* = 0.008, Fo: *P* < 0.001) had significant effects on prey consumption although the effects varied (Fig 2A_1_ and 2B_1_).

### Predation of prey species combinations

#### Invasive plus native prey: Bt or Fo plus Ag

Adult ladybirds readily attacked both invasive species in the presence of Ag: up to 80% of predator guts contained Bt and 60% contained Fo, without a significant temporal trend (Bt: *P* = 0.393, Fo: *P* = 0.729; Fig 1A_2_ and 1B_2_). In the presence of Ag, the percentage of predation on Bt over 36 h was 24.5% higher than on Fo (Paired *t*-test, *t* = 5.605, df = 6, *P* = 0.001; Fig 1A_2_ and 1B_2_). The maximum amount of invasive prey consumed over 36 h followed similar dynamics without a significant temporal trend (Bt: *P* = 0.559, Fo: *P* = 0.948; Fig 2A_2_ and 2B_2_), but the predator consumed 4.1–46.0% more Bt than Fo over this period (Paired *t*-test, *t* = 3.30, df = 3, *P* = 0.046; Fig 2A_2_ and 2B_2_).

#### Invasive prey species paired: Bt plus Fo

Adult ladybirds readily attacked both invasive species when they were encountered simultaneously: 90% of predators contained Bt DNA and 80% contained Fo DNA, with some decrease in Bt detected 8 h after release but this reduction was not significant (Bt: *P* = 0.463, Fo: *P* = 0.117; Fig 1A_3_ and 1B_3_). The dynamics over 36 h was similar, but predation was 6.1% higher on Bt than on Fo (Paired *t*-test, *t* = 3.286, df = 6, *P* = 0.017; Fig 1A_3_ and 1B_3_). The maximum amount of prey adults found over 36 h differed, but followed similar dynamics. For Bt, a maximum of about 1.19 adults was detected 4 h after release, compared to 0.59 Fo adults (Fig 2A_3_ and 2B_3_). Time after predator release had no significant effect on Bt consumption (*P* = 0.283) but a marginally significant one on Fo consumption (*P* = 0.052; Fig 2A_3_ and 2B_3_). Moreover, 9.2–58.5% more Bt was consumed over 36 h compared to Fo (Paired *t*-test, *t* = 3.080, df = 6, *P* = 0.022; Fig 2A_3_ and 2B_3_).

#### Three prey species together: Bt plus Fo plus Ag

Adult ladybirds readily attacked both invasive species even in this treatment. Up to 80% of predators contained invasive prey DNA, with some decrease 36 h after release but this reduction was not significant (Bt: *P* = 0.551, Fo: *P* = 0.318; Fig 1A_4_ and 1B_4_). The dynamics of percentage of predation on both invasive prey species over 36 h was similar (Fig 1A_4_ and 1B_4_). The maximum amount of prey consumed over 36 h differed between species but followed similar dynamics. For Bt, the maximum amount of prey was about 0.28 adults after 36 h, while that of Fo was 0.16, significantly less (Paired *t*-test, *t* = 9.257, df = 6, *P* < 0.001; Fig 2A_4_ and 2B_4_). Time after *H*. *axyridis* release had no significant effect on the consumption of either invasive prey (Bt: *P* = 0.324, Fo: *P* = 0.388).

#### Summary of prey consumption by *H*. *axyridis*

**Percentage of predation:** Neither prey species combination (*P* = 0.238) nor time (*P* = 0.253) had any significant effect on the percentage of *H*. *axyridis* preying on Bt, but the dynamics of predation were affected (Table 2; Fig 1). However, presence of the native Ag did not influence predation on Bt in any combination (*P* = 0.111–0.457; Fig 1).

**Table 2:** Two-way ANOVA (treatment and time after predator release) to compare the percentage of predation and number of prey adults consumed by *Harmonia axyridis* on *Bemisia tabaci* (Bt) or *Frankliniella occidentalis* (Fo).

Species	Source of variation	df	MS	*F*	*P*	LSD test^α^
Percentage of predator individuals that consumed Bt or Fo						
Bt	Treatments	3	0.145	1.440	0.238	n.s.
	Time	6	0.134	1.335	0.253	n.s.
	Error	74	0.101			
Fo	Treatments	3	1.370	10.936	0.000	Fo>Fo-Bt = Fo-Bt-Ag>Fo-Ag
	Time	6	0.178	1.422	0.218	n.s.
	Error	74	0.125			
Number of Bt or Fo adults detected in predator gut						
Bt	Treatments	3	2.842	14.991	0.000	Bt-Fo>Bt-Ag>Bt-Fo-Ag, Bt<Bt-Fo, Bt = Bt-Ag, Bt = Bt-Fo-Ag
	Time	6	0.490	2.585	0.018	T2<T8, T2 = T1 = T4 = T12 = T24 = T36, T8 = T1 = T4 = T12 = T24 = T36
	Error	378	0.190			
Fo	Treatments	3	4.965	33.057	0.000	Fo = Fo-Bt>Fo-Ag>Fo-Bt-Ag
	Time	6	0.456	3.073	0.007	T8>T36, T8 = T1 = T2 = T4 = T12 = T24, T36 = T1 = T2 = T4 = T12 = T24
	Error	350	0.150			

Bt alone (Bt), or with Ag (Bt-Ag), or with Fo (Bt-Fo), or in combination with Fo and Ag (Bt-Fo-Ag), and Fo alone (Fo), or with Ag (Fo-Ag), or with Bt (Fo-Bt), or in combination with Bt and Ag (Fo-Bt-Ag) were tested. Predators tested 1, 2, 4, 8, 12, 24 and 36 h (T1, T2, T4, T8, T12, T24 and T36, respectively) after release.

^α^ Differences based on the LSD (least significant difference) test (*P* < 0.05).

Prey species combination had a larger effect on Fo (*P* < 0.001) than on Bt (*P* = 0.238) predation by *H*. *axyridis* (Table 2). Whereas most predators consumed Fo (86.7–100.0%), the presence of Ag decreased predation significantly (60.0–86.7%; Paired *t*-test, *t* = 6.540, df = 6, *P* = 0.001; Table 2; Fig 1B_1_ and 1B_2_). Likewise, with three species available, fewer *H*. *axyridis* consumed Fo than when only Fo was available (*t* = 4.805, df = 6, *P* = 0.003; Table 2; Fig 1B_1_ and 1B_4_).

**Prey consumption:** There were significant effects of prey species combination (*P* < 0.001) and time after predator release (*P* = 0.018) on Bt consumption by *H*. *axyridis* (Table 2 and S1 Table; Fig 2A_1_–2A_4_). The highest Bt consumption occurred when Bt was together with Fo (0.33–1.19 adults), followed by the Bt-Ag combination (0.23–0.44 adults). Bt consumption was the lowest (0.16–0.28 adults) when all three species were present. Thus, the presence of Fo triggered an increase in Bt consumption by *H*. *axyridis* (Table 2; Fig 2A_1_–2A_4_), whereas no such effect was caused by Ag (*P* = 0.455; Fig 2A_1_ and 2A_2_).

There were also significant effects of prey species combination (*P* < 0.001) and time after predator release (*P* = 0.007) on Fo consumption (Table 2 and S1 Table; Fig 2B_1_–2B_4_). *H*. *axyridis* adults consumed more Fo when this was the only prey available (0.16–0.55 adults) or when present with Bt (0.18–0.59 female adults), followed by Fo plus Ag (0.15–0.42 female adults); consumption was least when all three prey were available together (0.08–0.16 female adults; Table 2; Fig 2B_1_–2B_4_).

## Discussion

The data in our present study showed unequivocally that the presence of native prey does not disrupt predation by a native predator on exotic prey. *H*. *axyridis* still attacked both invasive species in the presence of (superfluous numbers of) native aphids. This was indicated by the results of both the paired and three-prey experiments in which ample prey remained at the end of all experiments, ruling out the possibility of preferred prey exhaustion.

Although Berkvens *et al*. warn about possible bias when laboratory-reared predators are used in preference experiments [51], this potential source of bias can also be ruled out because we used field-collected *H*. *aryridis* adults. Because other species of whiteflies (*e*.*g*. *T*. *vaporariorum*) [52] and thrips (*e*.*g*. *Th*. *tabaci*, *F*. *intonsa*) [53] are native to China, it seems likely the predator may have pre-adapted to these prey types. Prey acceptance and nutritional value are often uncorrelated in coccinellids [54]. However, *H*. *axyridis* is highly polyphagous, and able to reproduce on many different kinds of prey species [51,54–56], including aphids [51] and whiteflies [7,9]. We observed no mortality of *H*. *aryridis* adults in any of the experimental cages, so we assume all three preys were nutritionally adequate [56]. However, this is not to infer that predator development and reproduction on these prey species will be equivalent [54,56].

The duplex qPCR assay developed here should be useful in tracking the dynamics of control efficacy of native predators on two of the most economically important invasive pest species, *B*. *tabaci* MEAM1 and *F*. *occidentalis*. In order to get reliable quantification, the primer half-life must be determined. Whereas the half-life of primer and probe sets for *B*. *tabaci* MEAM1 in *Propylea japonica* (Thunberg) is known [9], DNA amplification in the *H*. *axyridis* gut is not, so the efficiency of the primers used here could have influenced our results. However, because both frequency of predation and quantification point to the same trends, we believe that the data are mutually supportive, even though strict quantification cannot be claimed, and thus the conclusion that *H*. *axyridis* prefers *B*. *tabaci* MEAM1 to *F*. *occidentalis* is valid.

Although there is no experimental evidence of differences in prey quality of these two species, the relative profitability of the two exotic prey species could differ because of their differences in size and escape abilities. Thrips are smaller, and they also have a higher surface:volume ratio, hence increasing the relative amount of indigestible chitin. Therefore, they are probably less profitable to the coccinellid predators than whiteflies. Additionally, there is a difference in prey vulnerability. *B*. *tabaci* MEAM1 eggs are laid on the abaxial surface of leaves and thus available to predators, while the eggs of *F*. *occidentalis* are inside the tender plant tissues, and are more protected. *F*. *occidentalis* nymphs can also jump, which makes them more difficult to catch than those of the immobile whitefly nymphs.

Why do *H*. *axyridis* adults continue to prey on inferior, novel prey in the presence of more preferred, native prey? One possible reason is nutritional complementation via dietary mixing [57], as observed in other invertebrate predators, including spiders [58] and carabids [59]. Predators under field conditions are usually hungry [60,61], thus a lack of discrimination, *i*.*e*. attacking whatever potential prey they find, may be effective due to simple parsimony. Coccinellids can display both a lack of discrimination (*e*.*g*. even consuming toxic prey) [62] and an ability to discriminate using kairomones [63]. Additional behavioral studies could clarify interactions with various prey types.

Indirect interactions between herbivores, mediated by natural enemies may vary in nature and time [64–69]. The effects of relative prey densities linked to landscape complexity and/or predator behavior, such as switching [66,70,71], could be developed in regards to the differences in predation between the two exotic species. Usually, these indirect interactions are positive for the prey at time scales shorter than the predator generation time because of shared predation pressure on multiple pests, but become negative at longer time scales because of the predator numerical response [65,72]. The lepidopteran invader, *Tuta absoluta* Meyrick had a similar effect on the local pest *B*. *tabaci* MED controlled by the mirid predator *Macrolophus pygmaeus* Rambur on tomato crops [73]. In our present study, time after predator release had no significant effect on the percentage of *H*. *axyridis* individuals preying on *B*. *tabaci* MEAM1 or *F*. *occidentalis*, although our time scale was short (36 h). In spite of this, time after predator release had significant effects on number of prey consumption with a reduced predation rate after eight hours in multiple treatments, which could be related to the feeding behavior of the ladybird beetle, *e*.*g*. ladybird beetles usually search actively during the day.

The results of this study indicate that certain invasive prey species can be incorporated into the prey range of this native predator even when preferred native prey are available. This is an example of biotic resistance to invasion (that is standard terminology in invasion ecology). Such resistance can be an important factor in the process of both plant [74] and vertebrate [10] invasions. Evidence of biotic resistance during invertebrate invasions has been found for other invasive pests, with signs of preadaptation by native predators [75–78]. Due to the increase of such invasions world-wide [79], quantification of such biotic resistance would greatly help our understanding, and managing invasions. *H*. *axyridis* is used as an effective control agent worldwide and may at the same time causing ecological damage [55]. Further work should be conducted so as to assess which conditions favor it as being an effective control agent (versus a potential pest). Moreover, larger scale studies to assess the potential of the native generalist predator to control biological invasions should be included.

## Supporting Information

S1 Table One-way ANOVA (same time after predator release among different treatments) evaluating effects on percentage of predation and number of prey adults consumed by *Harmonia axyridis* on *Bemisia tabaci* MEAM1 (Bt) or *Frankliniella occidentalis* (Fo). (DOC)
