# Supplementary material for: Presence of Native Prey Does Not Divert Predation on Exotic Pests by Harmonia axyridis in Its Indigenous Range
Source: PLoS One. 2016 Jul 8;11(7):e0159048. doi: 10.1371/journal.pone.0159048 (PMC4938216; doi:10.1371/journal.pone.0159048)
Supplement: S1 Table — (DOC) [file pone.0159048.s001.doc]

**SI Table.** **One-way ANOVA (same time after predator release among different treatments) evaluating effects on percentage of predation and number of adults consumed by *Harmonia axyridis* on *Bemisia tabaci* MEAM1 (Bt) or *Frankliniella occidentalis* (Fo).**

| Species | Time after predator release (h) | Source of variation | | df | | MS | | *F* | *P* | | LSD test^α^ | |
| --- | --- | --- | --- | --- | --- | --- | --- | --- | --- | --- | --- | --- |
| Percentage of predator individuals that consumed Bt or Fo |  |  |  | | |  | |  | |  | |  |
| Bt | 1 | Between treatment | | 3 | | 33.333 | | 1.000 | 0.441 | | n.s. | |
|  |  | Within treatment | | 8 | | 33.333 | |  |  | |  | |
|  | 2 | Between treatment | | 3 | | 44.444 | | 0.190 | 0.900 | | n.s. | |
|  |  | Within treatment | | 8 | | 233.333 | |  |  | |  | |
|  | 4 | Between treatment | | 3 | | 177.778 | | 2.667 | 0.119 | | n.s. | |
|  | |
|  |  | Within treatment | | 8 | | 66.667 | |  |  | |  | |
|  | 8 | Between treatment | | 3 | | 133.333 | | 4.000 | 0.052 | | n.s. | |
|  |  | Within treatment | | 8 | | 33.333 | |  |  | |  | |
|  | 12 | Between treatment | | 3 | | 33.333 | | 0.100 | 0.958 | | n.s. | |
|  |  | Within treatment | | 8 | | 333.333 | |  |  | |  | |
|  | 24 | Between treatment | | 3 | | 122.222 | | 1.222 | 0.363 | | n.s. | |
|  |  | Within treatment | | 8 | | 100.000 | |  |  | |  | |
|  | 36 | Between treatment | | 3 | | 133.333 | | 1.333 | 0.330 | | n.s. | |
|  |  | Within treatment | | 8 | | 100.000 | |  |  | |  | |
| Fo | 1 | Between treatment | | 3 | | 755.556 | | 1.619 | 0.260 | | n.s. | |
|  |  | Within treatment | | 8 | | 466.667 | |  |  | |  | |
|  | 2 | Between treatment | | 3 | | 122.222 | | 0.611 | 0.627 | | n.s. | |
|  |  | Within treatment | | 8 | | 200.000 | |  |  | |  | |
|  | 4 | Between treatment | | 3 | | 1066.667 | | 32.000 | 0.000 | | Fo=Fo-Bt>Fo-Bt-Ag>Fo-Ag | |
|  |  | Within treatment | | 8 | | 33.333 | |  |  | |  | |
|  | 8 | Between treatment | | 3 | | 566.667 | | 4.250 | 0.045 | | Fo=Fo-Bt-Ag>Fo-Ag, Fo-Ag=Fo-Bt, Fo=Fo-Bt=Fo-Bt-Ag | |
|  |  | Within treatment | | 8 | | 133.333 | |  |  | |  | |
|  | 12 | Between treatment | | 3 | | 844.444 | | 3.167 | 0.085 | | n.s. | |
|  |  | Within treatment | | 8 | | 266.667 | |  |  | |  | |
|  | 24 | Between treatment | | 3 | | 488.889 | | 1.222 | 0.363 | | n.s. | |
|  |  | Within treatment | | 8 | | 400.000 | |  |  | |  | |
|  | 36 | Between treatment | | 3 | | 122.222 | | 0.407 | 0.752 | | n.s. | |
|  |  | Within treatment | | 8 | | 300.000 | |  |  | |  | |
| Number of Bt or Fo adults detected in predator gut |  |  | |  |  | |  | | |  |  | |
| Bt | 1 | Between treatment | | 3 | | 0.247 | | 1.644 | 0.190 | | n.s. | |
|  |  | Within treatment | | 55 | | 0.150 | |  |  | |  | |
|  | 2 | Between treatment | | 3 | | 1.443 | | 6.549 | 0.001 | | Bt<Bt-Ag=Bt-Fo, Bt-Fo>Bt-Fo-Ag, Bt-Ag=Bt-Fo-Ag, Bt=Bt-Fo-Ag | |
|  |  | Within treatment | | 50 | | 0.220 | |  |  | |  | |
|  | 4 | Between treatment | | 3 | | 0.917 | | 4.595 | 0.006 | | Bt=Bt-Fo-Ag<Bt-Fo, Bt-Ag=Bt-Fo, Bt=Bt-Ag=Bt-Fo-Ag | |
|  |  | Within treatment | | 52 | | 0.200 | |  |  | |  | |
|  | 8 | Between treatment | | 3 | | 0.334 | | 2.885 | 0.044 | | Bt-Fo>Bt-Fo-Ag, Bt=Bt-Ag=Bt-Fo, Bt=Bt-Ag=Bt-Fo-Ag | |
|  |  | Within treatment | | 54 | | 0.116 | |  |  | |  | |
|  | 12 | Between treatment | | 3 | | 0.736 | | 3.797 | 0.016 | | Bt=Bt-Ag=Bt-Fo>Bt-Fo-Ag | |
|  |  | Within treatment | | 49 | | 0.194 | |  |  | |  | |
|  | 24 | Between treatment | | 3 | | 0.562 | | 2.499 | 0.070 | | n.s. | |
|  |  | Within treatment | | 51 | | 0.225 | |  |  | |  | |
|  | 36 | Between treatment | | 3 | | 0.241 | | 1.231 | 0.309 | | n.s. | |
|  |  | Within treatment | | 49 | | 0.196 | |  |  | |  | |
| Fo | 1 | Between treatment | | 3 | | 0.390 | | 2.351 | 0.083 | | n.s. | |
|  |  | Within treatment | | 50 | | 0.166 | |  |  | |  | |
|  | 2 | Between treatment | | 3 | | 1.737 | | 7.783 | 0.000 | | Fo=Fo-Bt>Fo-Ag=Fo-Bt-Ag | |
|  |  | Within treatment | | 50 | | 0.223 | |  |  | |  | |
|  | 4 | Between treatment | | 3 | | 1.070 | | 5.540 | 0.002 | | Fo=Fo-Bt>Fo-Ag=Fo-Bt-Ag | |
|  |  | Within treatment | | 48 | | 0.193 | |  |  | |  | |
|  | 8 | Between treatment | | 3 | | 0.959 | | 10.360 | 0.000 | | Fo=Fo-Bt>Fo-Ag=Fo-Bt-Ag | |
|  |  | Within treatment | | 49 | | 0.093 | |  |  | |  | |
|  | 12 | Between treatment | | 3 | | 0.946 | | 7.050 | 0.001 | | Fo=Fo-Ag=Fo-Bt>Fo-Bt-Ag | |
|  |  | Within treatment | | 46 | | 0.134 | |  |  | |  | |
|  | 24 | Between treatment | | 3 | | 0.826 | | 8.595 | 0.000 | | Fo>Fo-Bt>Fo-Bt-Ag, Fo-Ag>Fo-Bt-Ag, Fo=Fo-Ag, Fo-Ag=Fo-Bt | |
|  |  | Within treatment | | 46 | | 0.096 | |  |  | |  | |
|  | 36 | Between treatment | | 3 | | 0.091 | | 0.706 | 0.554 | | n.s. | |
|  |  | Within treatment | | 43 | | 0.129 | |  |  | |  | |

Bt alone (Bt), or with Ag (Bt-Ag), or with Fo (Bt-Fo), or in combination with Fo and Ag (Bt-Fo-Ag). Fo alone (Fo), or with Ag (Fo-Ag), or with Bt (Fo-Bt), or in combination with Bt and Ag (Fo-Bt-Ag). Predators tested 1, 2, 4, 8, 12, 24 and 36 h after release.

^α^ Differences based on the LSD (least significant difference) test (*P* < 0.05).
